# Lisfranc fracture-dislocation precipitating acute Charcot arthopathy in a neuropathic diabetic foot: a case report

**DOI:** 10.1186/1757-1626-1-290

**Published:** 2008-10-31

**Authors:** Joey Yeoh, Kenneth Ross Muir, Ajith Munasinghe Dissanayake, Wendy Yu Tzu-Chieh

**Affiliations:** 1 Department of Endocrinology and Diabetes, Centre for Clinical Research and Effective Practice, Room 33, Support Building, Middlemore Hospital, Auckland, New Zealand; 2 Department of Orthopaedic Surgery, Middlemore Hospital, Auckland, New Zealand

## Abstract

The Lisfranc injury is relatively uncommon yet remains popular in the literature due to its variable causative mechanisms and subtleties in radiographic features despite its potential for disabling long term outcomes if treatment is inadequate, inappropriate or delayed. These injuries are especially pertinent in diabetic patients, especially those with neuropathy, since they are more common, can lead to Charcot neuropathic joint, ulcers and have different causative mechanisms compared to the general population. We describe the case of a neuropathic diabetic patient who presented with a Lisfranc injury which precipitated the development of acute Charcot arthropathy in the right foot. The case serves to illustrate several salient points about the Lisfranc joint and related injuries in diabetic patients.

## Introduction

Lisfranc fracture-dislocations/injuries involve the Lisfranc joint which is named after the 18^th ^century French field surgeon, Jacques Lisfranc de Saint-Martin [1]. Although the tarsometatarsal joint (TMT) was described by Jacques Lisfranc as an amputation level, he did not describe Lisfranc injuries [2]. Lisfranc injuries can be bony, ligamentous or a combination of both and can result from direct or indirect and high-velocity or low-velocity mechanisms [3,4]. In non-diabetic patients, high-velocity indirect mechanisms which cause axial loading or rotation on a plantarflexed foot are most common [3].

In diabetic patients, especially those with peripheral neuropathy, Lisfranc injuries can result from minimal or no trauma and can be a precipitant and/or manifestation of Charcot arthropathy [3,4]. Clinical symptoms include soft tissue swelling, increased warmth, inability to weight bear and significant pain [1,3,4]. Treatment depends on the severity and exact nature of the Lisfranc injury, ranging from conservative immobilization by casting to open reduction and internal fixation (ORIF) with Kirschner wires (K-wires), transarticular absorbable screws or plate and screws [5].

## Case presentation

A 56 year old Caucasian male presented to the ward via the podiatrist with a 5 day history of a hot, swollen and painful right foot leading to the inability to weight bear over the past 2 days. The patient had slipped down 2 steps while walking down a short staircase at home 6 days prior. The podiatrist had been seeing him regularly in the outpatient diabetic foot clinic for his left 1^st ^metatarsal head plantar neuropathic ulcer. He had been put into a left total contact cast (TCC) for the past 1 month following debridement and dressing which resulted in good healing of the ulcer.

The patient is a known Type 1a diabetic since 1982 with established microvascular complications. He has end stage renal failure (ESRF) from diabetic nephropathy and has been on renal replacement therapy for the past 5 years. He has bilateral stable proliferative retinopathy with previous multiple laser photocoagulations and residual visual acuity of 6/36 right and 6/9 left. He also suffers from peripheral neuropathy with mild plantar neuropathic ulcers affecting the right and left feet in the past. He also has dyslipidaemia and hypertension.

There is a family history of Type 2 diabetes in the father and grandmother. He does not smoke or consume alcohol and works as a storeman. His list of current medications include; insulin Lispro (Humalog) 12 units three times a day (TDS) pre-meals subcutaneously (S/C), insulin Glargine 42 units once daily (OD) S/C, Metoprolol succinate 23.75 mg OD, Cilazapril monohydrate 2.5 mg OD, Erythropoietin beta 6000 units thrice weekly S/C, Simvastatin 40 mg OD, calcitriol 0.5 micrograms thrice weekly, calcium citrate 1200 mg once weekly, Aspirin 100 mg OD and Omeprazole 20 mg OD. He weighs 101.5 kilograms (kg) and stands 1.85 metres (m) tall, making the body mass index (BMI) 29.7 kg/m^2^.

Cardiovascular, respiratory and abdominal examination revealed no significant abnormalities. Neurological examination using Semmes-Weinstein 10 g (5.07) monofilament revealed loss of neuroprotective sensation bilaterally. There was also loss of proprioception and vibration sense bilaterally. Peripheral pulses were normal. The right foot was noted to be oedematous, erythematous and was tender to palpation around the dorsal midfoot. The cutaneous temperature of the right foot was 4°C higher than the left. The recently healed left plantar ulcer was also seen.

**Figure 1:**
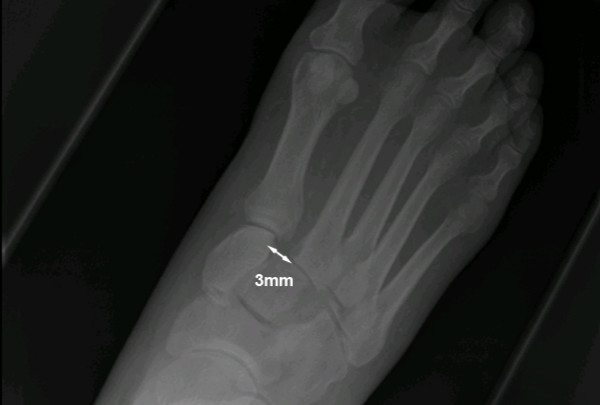
**Dorsoplantar weight-bearing film of the right foot**. The radiograph shows a 3 mm lateral displacement of the 2^nd ^metatarsal and a mild 2 mm lateral displacement of the first metatarsal consistent with a homolateral (Type B2) Lisfranc injury.

**Figure 2:**
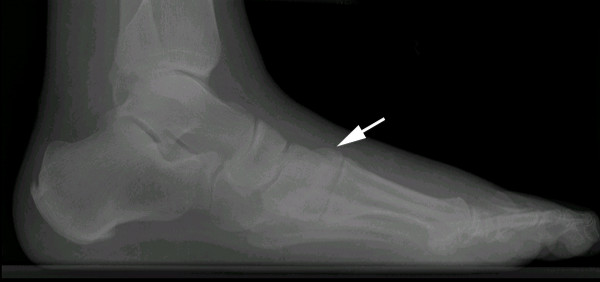
**Lateral weight-bearing film of the right foot**. This radiograph shows the characteristic "step-off" point (arrow) caused by dorsal displacement of the 2^nd ^metatarsal relative to the medial cuneiform.

He had bilateral feet and ankle radiography, including weight-bearing films. The left foot and ankle films were unremarkable, showing no overt neuropathic arthropathy or Lisfranc joint problems. The right foot dorsoplantar weight-bearing film showed malalignment with lateral displacement of the 2^nd ^metatarsal by 3 mm and a mild 2 mm lateral displacement of the first metatarsal consistent with a homolateral (Type B2) Lisfranc injury (Figure 1). The lateral weight-bearing film showed the classical "step-off" point (Figure 2).

**Figure 3:**
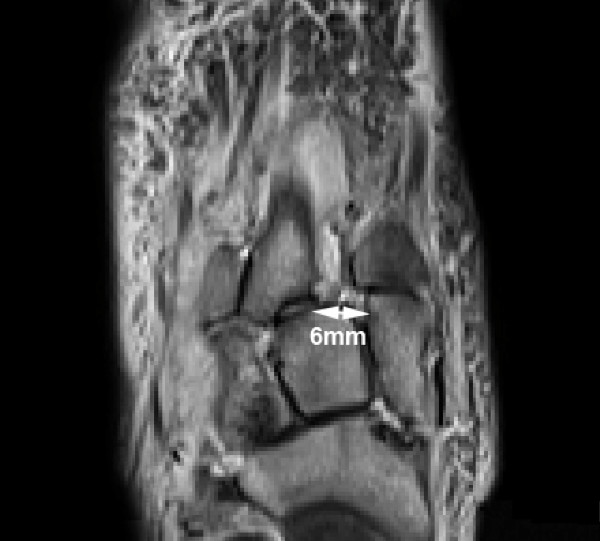
**STIR sequence oblique axial view of the right foot**. This shows the 6 mm lateral displacement of the 2nd and 3rd metatarsals and a completely ruptured Lisfranc ligament which should usually be seen crossing the 1^st ^metatarsal space obliquely. The ruptured ligament would explain why the typical "fleck sign" was absent on the plain radiograph as a ruptured ligament will not avulse the lateral border of the medial cuneiform or the medial border of the 2^nd ^metatarsal base.

Magnetic resonance imaging (MRI) was subsequently done. Axial and coronal T1 and Short T1 inversion recovery (STIR) sequences were undertaken. The left foot did not reveal any significant findings while the right foot scan showed high STIR signal within all the muscles of the flexor compartment, oedematous thickening of the interosseous muscles and low T1 with high STIR signal within the 2^nd ^and 3^rd ^metatarsal bases and tarsal bones consistent with acute (Stage 1) Charcot changes. A 6 mm lateral displacement of the 2^nd ^and 3^rd ^metatarsals was also detected. Oblique axial STIR views revealed a complete rupture of the Lisfranc ligament (Figure 3).

He was treated initially with bedrest and analgesia followed by TCC for the right Lisfranc injury and acute Charcot. A prefabricated pneumatic walking brace (PPWB) was used for the left leg to prevent unequal distribution of pressure and thus possibility of further ulceration. We discharged the patient with the plan for the TCC to remain for the next 4 – 6 months (with 2 – 3 weekly re-casting) depending on resolution of the acute phase and achievement of anatomic alignment, followed by bracing.

## Discussion

Important learning points about the Lisfranc joint and associated injuries in diabetic patients can be derived from this case. Firstly, although Lisfranc injuries are rare, accounting for only 1% of orthopaedic injuries, they are more common in the diabetic population [3,4,6]. However, the exact prevalence and incidence of Lisfranc injuries in diabetics is currently unknown [3,4]. Secondly, Lisfranc injuries in diabetic patients frequently occur following minimal trauma (such as slipping or tripping off a step or a curb) or no obvious trauma at all [3,4]. Diabetic patients with loss of neuroprotective sensation are at significantly higher risk of developing Lisfranc injuries without antecedent trauma because of the increased likelihood of repeated unperceived microtrauma similar to the neurotraumatic theory of Charcot pathogenesis [3,4,7]. Thirdly, complete rupture of the Lisfranc ligament usually requires a significant amount of force, but in diabetic patients only minimal force may be required secondary to calcification of ligaments, repeated microtrauma and poor healing which all result in loss of tensile strength and increased susceptibility to rupture [8].

Fourthly, the Lisfranc joint is the most common site affected in Charcot arthropathy of the foot and accounts for 45% of cases [7]. In fact, the medial column of the TMT joint is where early Charcot changes usually begin [7]. Therefore, Lisfranc injuries and the resultant architectural instability coupled with pre-existing neuropathy can precipitate the Charcot process or alternatively be the first sign of an underlying Charcot process [3,4,7]. In addition, Lisfranc injuries and acute Charcot foot may be clinically indistinguishable [3,4,7]. It is therefore essential to look closely for Charcot changes on imaging in diabetics with Lisfranc injuries [3].

Lastly, Lisfranc injuries in diabetics that do not result in an acute Charcot process may still lead to unequal distribution of pressure in the affected foot and this, coupled with poor wound healing and the propensity for infections inherent in diabetics may lead to the development of plantar ulcers if patient presentation and diagnosis is delayed [3,4,6]. One final but crucial point regarding imaging of Lisfranc injuries in diabetic patients is that radiographic findings are frequently subtle, easily missed and may not properly represent the underlying degree of diastasis [4,6].

This is aptly demonstrated by the case presentation where the subluxation/dislocation of the 2^nd ^metatarsal on MRI was 6 mm compared to 3 mm on plain radiography. In fact, up to 10% of subtle injuries such as those associated with minor or no trauma in diabetics can spontaneously reduce and thus be missed if weight-bearing radiographic views are not obtained [6]. Therein lies the value of MRI examination which allows optimal evaluation of malalignment in the midfoot, with the oblique axial plane allowing visualization of the entire length of the Lisfranc ligament [6].

## Conclusion

Lisfranc injuries are relatively uncommon injuries affecting the midfoot which frequently have different characteristics in the diabetic population. Understanding these differences allow us to diagnose, treat and monitor the resolution of the injuries effectively and therefore prevent poor anatomic alignment which can have particularly dire consequences in diabetic patients.

## Consent

Written informed consent was obtained from the patient for publication of this case report. A copy of the written consent is available for review by the Editor-in-Chief of this journal.

## Competing interests

The authors of this case report declare that they have no competing or financial interests that could influence the preparation of the manuscript.

## Authors' contributions

KRM collected the details of the case. KRM and WYTC were responsible for the literature search. JY was responsible for the literature review and preparation of the first draft of the manuscript. AMD managed the patient during in-patient hospital stay. All the authors read and approved the final manuscript.
